# How efficacious is the combination of substitute bone graft with autogenous bone graft in comparison with substitute bone graft alone in the horizontal bone gain? A systematic review and meta-analysis

**DOI:** 10.4317/jced.59087

**Published:** 2022-08-01

**Authors:** Jonathan Meza-Mauricio, Camila-Pinheiro Furquim, Leonardo-Delfino dos Reis, Marlon-Marx-Hilariano Maximiano, Gerardo Mendoza-Azpur, Francisco-Wilker-Mustafa-Gomes Muniz, Giulio Rasperini, Marcelo Faveri

**Affiliations:** 1 Department of Periodontology, School of dentistry Universidad Cientifica del Sur Lima Peru; 2 Department of Periodontology and Oral Implantology, Dental Research Division, University of Guarulhos, SP, Brazil; 3 Department of Periodontology, Graduate Program in Dentistry, Federal University of Pelotas, Pelotas, Brazil; 4 Department of Biomedical, Surgical and Dental Sciences, Foundation IRCCS Ca’ Granda Polyclinic, Milan, Italy

## Abstract

**Background:**

A systematic review (SR) was conducted to answer the following focused question based on PICO strategy: In patients who were submitted to horizontal guided bone regeneration, “how efficacious is the combination of substitute bone graft with autogenous bone graft in comparison with substitute bone graft alone, in terms of bone gain?”

**Material and Methods:**

MEDLINE (PubMed), Scopus, Embase, Web of Science databases were searched, and hand searches were made up to June 2021, to find randomized clinical trials comparing the clinical effects of autogenous bone graft + substitute bone graft versus substitute bone graft alone in the treatment of horizontal guided bone regeneration.

**Results:**

Four trials representing 109 individuals were included. All studies included in this SR used allogeneic bone graft. The meta-analysis did not show any statistically significant difference between the groups, for horizontal bone gain at a distance of 0 mm (MD: -0.46; 95%CI: -1.03 – 0.11) or at a distance of 4 to 5 mm from the top of the crestal alveolar ridge (MD: 0.17; 95%CI: -1.08 – 1.42).

**Conclusions:**

Within limitations of this systematic review, it was concluded that the addition of autogenous bone graft to the allogeneic bone graft did not significantly increase the quantity of regenerated bone.

** Key words:**Bone graft, bone regeneration, allograft.

## Introduction

Osseointegrated dental implants are an ideal alternative solution to replacing missing teeth; however, their successful placement is directly related to the quantity and quality of bone in edentulous areas (1). Tooth removal is often followed by a bone remodeling process leading to gradual reduction in horizontal and vertical bone ridge height (2). Bone resorption primarily occurs in the buccal aspect and increases over time (3). During the first year, loss of teeth results in a 25% reduction in bone width and a decrease of 4 mm in vertical bone height (4), and these changes could negatively affect the esthetic results of a final rehabilitation, irrespective of whether traditional rehabilitation or dental implant placement is performed (5). If an appropriate 3D positioning of implants cannot be achieved in the residual bone, ridge augmentation should be performed (6). Several techniques are now being used [for this purpose] such as distraction osteogenesis, ridge splitting, autogenous bone (AB) block, and guided bone regeneration (GBR) (7).

Augmentation using GBR has become a major treatment option to provide optimal bone support for osseointegrated dental implants. Simple defects, including dehiscence and fenestration defects were initially treated with GBR (8). Furthermore, GBR has been used for horizontal and vertical ridge augmentations and the success rate of implant treatment in the area restored by the GBR technique has been shown to similar to that of implant treatment in the healthy native bone region (9).

AB is considerate the gold standard graft material for GBR because of its osteogenic, osteoinductive, and osteoconductive properties (10). AB is commonly harvested from the ramus and symphysis (11,12). Considering its limitations, autologous grafts may need a second surgical site to enable their harvesting, which increases patient’s morbidity, pain or discomfort, and other complications related to increased surgical time and invasiveness of the procedure (10). As an alternative to autogenous graft, various bone substitutes, including xenografts, alloplastic grafts, and allografts have been used to GBR (13,14). However, some researchers have advocated the necessity of mixing AB with the various bone substitutes in order to combine the osteogenic and osteoinductive growth factors of autogenous bone with the osteoconductive properties of a bone substitute (15-17).

To date, there still is no consensus about the need to combine substitute graft material with AB graft, but the addition of AB graft could increase bone formation in comparison with allogenic graft alone (18). Therefore, the present systematic review was conducted to answer the following focused question based on PICO strategy: In patients who were submitted to horizontal guided bone regeneration, how efficacious is the combination of substitute bone graft with autogenous bone graft in comparison with substitute bone graft alone, in terms of bone gain? 

## Material and Methods

-Protocol and registration

This systematic review was conducted in accordance with the Transparent Reporting of Systematic Reviews and Meta-Analysis – PRISMA Statement (19). Its protocol was registered on INPLASY (registration number 202180109) and is available in full on the inplasy.com (https://inplasy.com/inplasy-2021-8-0109/) platform.

-Focused question 

In patients who were submitted to horizontal guided bone regeneration, how efficacious is the combination of substitute bone graft with autogenous bone graft in comparison to substitute bone graft alone, in terms of bone gain?

-Eligibility criteria

The inclusion criteria were based on the PICOS strategy 20. Only studies meeting the following criteria were included:

•Inclusion criteria (PICOS)

(P)opulation: Patients with horizontal atrophic alveolar ridge in need of horizontal guided bone regeneration prior to dental implant installation.

(I)ntervention: Bone augmentation using guide bone regeneration. This procedure needed to have been performed with particulate materials, such as autologous bone chips, and/or osteoconductive materials, such as allografts, xenografts, or alloplastic bone substitute materials.

(C)omparison: Substitute bone graft + autogenous bone graft vs substitute bone graft alone.

(O)utcome: Horizontal bone gain determined by any radiographic analysis or any other method (primary outcome variable), graft resorption, and histological findings and patient-reported outcome measurements (PROMs) (pain, discomfort, satisfaction, etc.) (secondary outcome variables).

(S)tudy design: Randomized clinical trial (RCTs).

Only studies that involved adult individuals (aged at least 18 years old) were included. No restriction on ethnicity or gender were imposed. No minimum number of individuals per group was established.

•Exclusion criteria

i. Studies that included individuals with systemic diseases or conditions that might compromise the guided bone regeneration procedure (e.g., diabetes).

-Search strategy

The MEDLINE (PubMed), Embase, Scopus, and Web of Science databases were searched up to June 2021 by two independent reviewers (J.M.M. and C.P.F.). The search was performed without restrictions on dates or language. The search strategy was applied as follows: PubMed: (“Horizontal ridge deficiencies”[All Fields] OR “horizontal alveolar ridge augmentation”[All Fields] OR “lateral ridge augmentation”[All Fields] OR “Lateral bone augmentation”[All Fields] OR “Horizontal bone augmentation”[All Fields] OR “horizontal Bone regeneration”[All Fields] OR “Horizontal ridge augmentation”[All Fields]) AND (“Bone graft”[All Fields] OR “Autologous bone”[All Fields] OR “Autogenous bone”[All Fields] OR “Bone substitute”[All Fields] OR “Allograft bone”[All Fields] OR “Allogenic bone”[All Fields] OR “Xenogeneic bone”[All Fields] OR “Alloplastic”[All Fields]). In addition, the grey literature in the System for Information on Grey Literature in Europe (http://www.opengrey.eu) and The New York Academy of Medicine Grey Literature Report (http://www.greylit.org) were electronically screened, as recommended by the high standards for systematic reviews (AMSTAR guideline) (21). Furthermore, hand searches of relevant primary sources related to the topic were performed in Clinical Implants Dentistry and Related Research, Journal of Clinical Periodontology, Clinical Oral Implants Research and Clinical Oral Investigations. Finally, the list of references of studies included were also hand searched to capture any potential additional records, as suggested by Greenhalgh and Peacock (22).

-Data collection, extraction and management

•Screening and selection of papers

Titles and abstracts were screened by two reviewers independently (J.M.M. and G.M.A). Full-text reports were obtained and reviewed independently for studies that seemed to meet the previously mentioned inclusion criteria. Kappa scores (Cohen’s ĸ coefficient) were used during full-text assessment to ensure eligibility and level of agreement between the reviewers. Disagreements were resolved by discussion and consulting a third reviewer (M.F).

•Data extraction

The studies that fulfilled the eligibility criteria were processed for data extraction, conducted by two independent researchers (M.M.H.M and L.R), using predefined spreadsheets. Disagreements were resolved by discussion with a third reviewer (M.F). In the event of missing data, a request was sent to the authors by e-mail. For each study selected, the following variables were collected: journal, name of author(s), year of publication, study design, intervention type, membrane, graft material, membrane fixation method, horizontal bone gain, mean of graft resorption, the percentage of autogenous bone graft in relation to substitute bone graft, histological findings, patient reported outcomes, number of patients (in each experimental group), and follow-up period.

•Risk of bias in individual studies 

Two reviewers (C.P.F and J.M.M) assessed the risk of bias in the studies selected, using the Cochrane risk-of-bias tool, RoB 2 (version 2, available at: https://www.riskofbias.info/welcome/rob-2-0-tool/current-version-of-rob-2). The authors of this systematic review decided to assess the result related to “assignment to intervention (the intention-to-treat effect)” and five domains were examined. Based on the answers to signaling questions and algorithms of this tool, each domain was judged as presenting “low risk of bias”, “some concerns relating to the risk of bias,” or “high risk of bias”. Studies were categorized as being at low risk of bias (all domains were at low risk of bias), high risk of bias (one or more domains were at high risk of bias), some concerns (if one or more domains had some concerns) (23). Disagreements were resolved by discussion, consulting a third researcher (G.R.).

•Data analyses and synthesis of the results

One author (F.W.M.G.M.) was responsible for statistical data collection and analysis. Two meta-analyses for horizontal bone gain were performed in the present study, as two distances from the top of the crest were considered: 1) zero mm and 2) 4-5mm. For both analyses, mean difference (MD) between baseline and the last follow-up period were calculated for each study and each experimental group. The 95% confidence intervals (95%CI) were also calculated for each study, and the level of significance established was *p*<0.05. One of the studies included presented data of sites with postoperative infection, but this information was not retrieved in the current analysis (24). Moreover, as both acellular dermal matrix and collagenous membrane were used for guided bone regeneration, subgroup analyses were considered in both meta-analyses.

The software Rev-Man (version 5.3 for Windows, The Cochrane Collaboration, Copenhagen) was used to perform both meta-analyses. Statistical heterogeneity between both groups was assessed using Cochran’s Q-test, with a threshold P-value of 0.1, and the inconsistency I2 test, in which values >50% were considered indicative of high heterogeneity. As the methodological characteristics differed among the studies included (some studies have used acellular dermal matrix and other used collagen membrane), both analyses were performed using a random effect model.

-Certainty of the evidence 

The overall quality of the evidence for each outcome assessed in the meta-analysis was rated using the Grading of “Recommendation Assessment, Development and Evaluation (GRADE)” according to its level of certainty: very low, low, moderate, and high (25). The GRADE approach integrates the intra-study risk of bias, unexplained heterogeneity or inconsistency, indirectness (adequacy to PICOS focused question), imprecision and other considerations (e.g., publication bias, magnitude of the effect, plausible confounding, and dose-response gradient).

## Results

-Study Selection 

**Figure 1:**
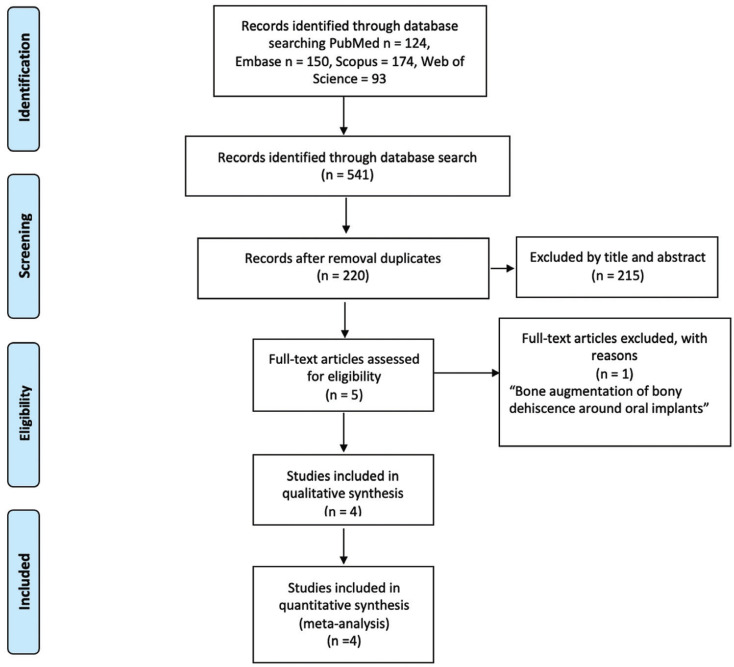
PRISMA flowchart of the manuscripts screened through the review process.

A total of 541 studies were detected using the search strategy. After removal of the duplicates, 220 records were screened for eligibility. Of these, 215 studies were excluded by title and abstract, and 5 full texts were accessed and evaluated. One study was excluded from this review because did not metal the eligibility criteria (bone augmentation of bony dehiscence around oral implants) (18). Therefore, 4 studies were included in the systematic review and meta-analyses (Fig. 1). The reviewers showed excellent agreement (Kappa ≥ 0.85).

-Study characteristics

The four RCTs (24,26-28) included were conducted between 2010 and 2020, and their main methodological characteristics are presented in Table 1. Two clinical studies were conducted in USA (24,28), and the others in different countries, namely Israel (27) and Iran (28). All clinical studies included in this systematic review used freeze-dried bone allograft. Therefore, the following group was evaluated: allogeneic bone graft + autogenous bone graft (test) versus allogeneic bone graft alone (control). A total of 109 (55 test and 54 control) individuals within the age-rang of 21 – 73 were included in this systematic review. The studies included in this review used different membranes to covered the bone graft material; namely, acellular dermal matrix (25,27) and collagen membrane (26,29). To fix these membranes, different approaches were used, such as: titanium tacks (24), bone screw (27), suture (in the horizontal mattress technique, sutures were anchored to the periosteum remaining on the bone in the apical region of the facial flap) (28). In only one clinical study, the membrane was not fixed (26). Finally, to evaluate the horizontal bone gain, cone-beam computerized tomography was used in three studies (24,26,28), and in one study, a modified digital caliper was used (31).

**Table 1:**
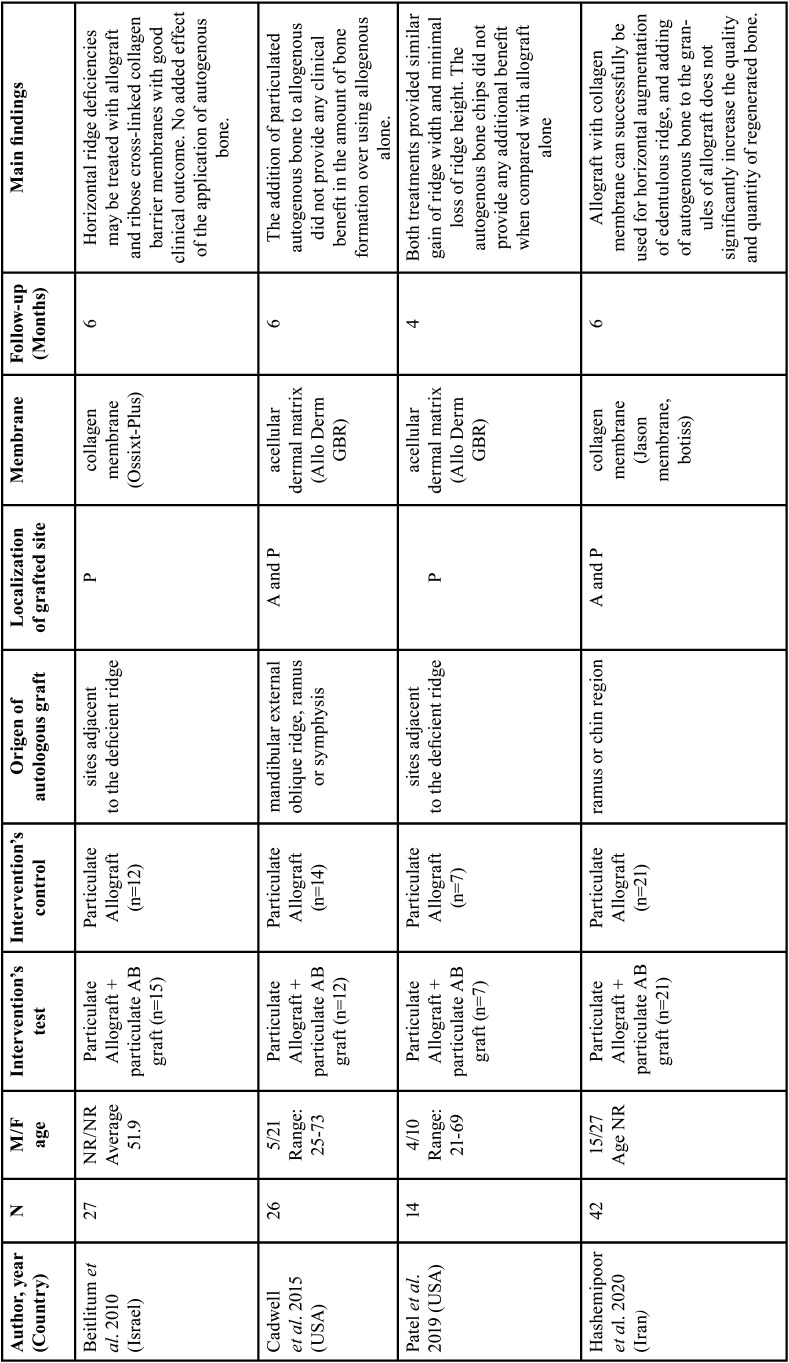
Main methodological characteristic and results of the studies included.

-Risk of bias of the individual studies

**Figure 2:**
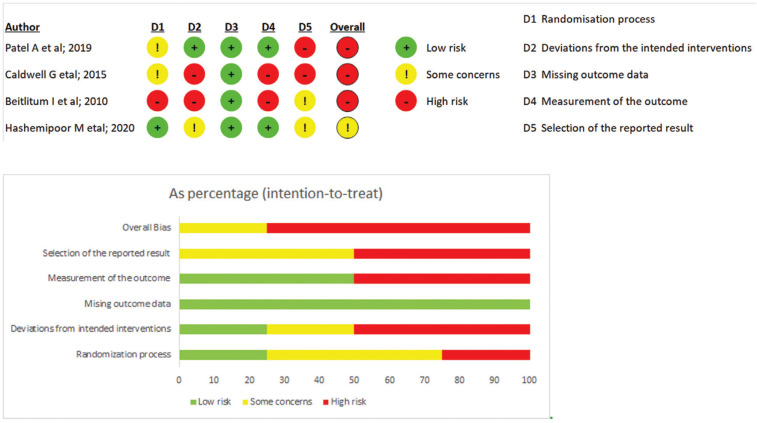
Summary of the risk of bias of the included studies in systematic review, according to Cochrane risk-of-bias tool, RoB 2. Plus sign indicates low risk of bias; minus sign indicates high risk of bias; exclamation mark indicates some concerns for the risk of bias.

All studies presented some problems with risk of bias. Relative to the randomization process, this was applied in only one study for allocation of participants; and no baseline differences between groups was shown (28). In the other studies, no information was found about the randomization process (26), the allocation sequence (26,27) and differences between experimental and control at baseline (24,26). In the second domain of the ROB 2 tool, all participants were aware of the treatment received because of the study context (It is was not possible to blind the participants because of the donor site used in the Experimental Group), However only one study described how caregivers and professionals delivering the interventions were blinded (27). In the study of Betilium *et al*., the same surgeon was responsible for taking the measurements, increasing the risk of bias for this outcome (26). In the selection of results reported, only one study was registered in a clinical trials database, and did so after conclusion of the study 28. A summary, elaborated by using a specific graphic tool, is presented in (Fig. 2).

Ratio of autogenous bone graft and allogeneic bone graft

The percentage of autogenous bone graft in relation to allogeneic bone graft was described in three studies 24,27,28. In two studies, the percentage was 50% of autogenous bone graft and 50% of allogeneic bone graft 24,28. Whereas in another study, the percentage of allograft was 70% and of autogenous bone 30% 27. To harvest the autogenous bone graft, different instruments, such as bone scrapers 26,27, ultrasonic bone saw 24, and rotary collecting bone instrument 28 were used in the studies.

-Clinical Results 

At baseline, the mean value of horizontal bone in Groups Allogeneic bone graft + Autogenous bone graft and Allogeneic bone graft alone were 3.82 ± 1.4 mm, 3.21 ± 1.2 mm (24); 3.53 ± 1.3 mm, 2.17 ± 0.58 mm (26); 3.2 ± 0.7 mm, 3.0 ± 0.6 mm (27); 1.9 ± 0.78 mm, 2.22 ± 1.02 mm (28) respectively. Only one study showed a statistically significant difference for the comparison between groups (26). The evaluation time reported in the selected studies varied widely. The evaluation time was 6 months in three studies (24,26,28) and 4 months in one study (27). After the surgical procedures, the mean of horizontal bone value in Groups Allograft bone + Autogenous bone graft and Allograft alone were 6.75 ± 1.22 mm, 6.25 ± 1.35 mm [28]; 7.13 ± 1.96 mm, 7.17 ± 1.28 mm (26); 6.7 ± 1.7 mm, 6.8 ± 1.9 mm 27; 4.3 ± 1.68 mm, 5 ± 1.15 mm (28), respectively. In terms of mean horizontal bone gain, all studies included in this systematic review found no statistically significant difference between groups (24,26-28).

Two studies included in this systematic review evaluated the dental implants placement after the bone augmentation. Hashemipoor *et al*., showed that only two patients in the Allogeneic bone graft group needed to receive complementary GBR in the implant insertion session due to insufficient quantity of bone regeneration (28). On the other hand, Beitlitum *et al*., showed that two patients in the Allogeneic bone graft group and 3 patients in the Allogeneic bone graft + Autogenous bone graft group needed to receive complementary GBR (26).

The mean rate of graft resorption between baseline and reentry was evaluated in two studies (24,27). In the study of Cadwel *et al*., the rate of resorption in Group Allogeneic bone graft + Autogenous bone was 0.68 ± 0.72 mm or 16.91% ± 17.10%, whereas for Group Allogeneic bone graft, it was 0.42 ± 0.60 mm or 11.12% ± 15.11% (*p*>0.05) 24. Furthermore, in the study of Patel *et al*., the mean rate of graft resorption was 2.9 ± 1.9 mm or 30%, and 2.3 ± 1.9 mm, or 25% for Group Allogeneic bone graft + Autogenous bone, and Group Allogeneic bone graft, respectively (*p*>0.05) (27).

-Histological findings 

The histological examination was performed in two clinical studies. Patel *et al*. showed that Group Allogeneic bone graft + Autogenous bone had mean values of 35% vital bone, 26% nonvital bone, and 39% trabecular space. While, Group Allogeneic bone graft had mean values of 39% vital bone, 24% nonvital bone, and 37% trabecular space. There were no statistically significant differences between groups (*p* >0.05) (27). The study of Hashemipoor *et al*. showed mean values of 46.07±6.34% new bone, 9.08±2.33% remaining graft particles and 44.83±6.55% soft tissue in Group Allogeneic bone graft + autogenous bone. While, Group Allogeneic bone graft alone showed mean values of 43.71±5.63% new bone, 9.86±2.16% remaining graft particles and 46.42±7.33% soft tissue. The difference between the two groups was not statistically significant (*p* >0.05) (28).

-Patient-reported outcome measures

The effect of guide bone regeneration modalities on PROMs could not be investigated, as these outcomes were not reported in any clinical trial.

-Synthesis of meta-analysis results

**Figure 3:**
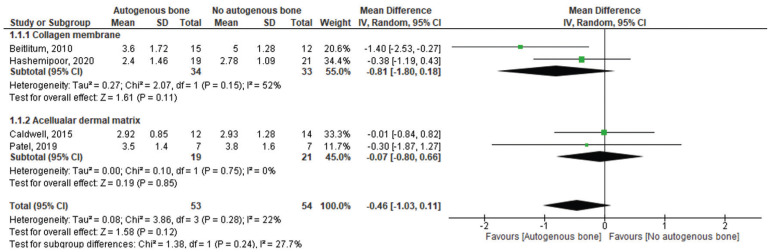
Forest plot for the horizontal bone gain at a distance of 0 mm from the crestal alveolar ridge autogenous bone graft + allogeneic bone graft vs, allogeneic bone graft alone. Acellular dermal matrix and collagenous membrane used for guided bone regeneration was used as a subgroup.

Figure 3 shows the meta-analysis for the values of horizontal bone gain at a distance of 0mm from the crestal alveolar ridge, which were included in four studies (24,26-28). No statistically significant difference between groups was detected (MD: -0.46; 95%CI: -1.03 – 0.11). For this analysis, low heterogeneity was detected (I2=22%, *P*=0.28). When the subgroup analysis was performed, a similar trend of results was detected to both collagen membrane (MD: -0.81; 95%CI: -1.80 – 0.18) and acellular dermal matrix (MD: -0.07; 95%CI: -0.80 – 0.66) (Fig. 3).

**Figure 4:**
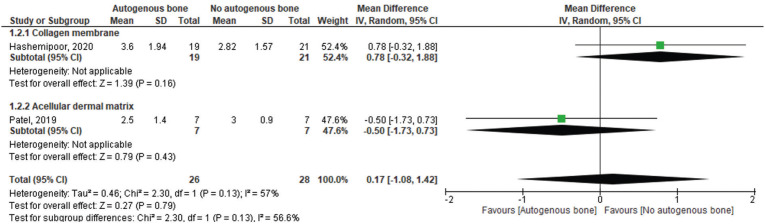
Forest plot for the horizontal bone gain at a distance of 4 to 5 mm from the crestal alveolar ridge autogenous bone graft + allogeneic bone graft vs allogeneic bone graft alone. Acellular dermal matrix and collagenous membrane used for guided bone regeneration was used as a subgroup.

Similar results were detected when Groups were compared for the horizontal bone gain values at a distance of 4 to 5 mm from the crestal alveolar ridge (MD: 0.17; 95%CI: -1.08 – 1.42). A higher heterogeneity was detected in this analysis (I2=57%, *P*=0.13) (Fig. 4). It is important to highlight that only two studies were included in this analysis (27,28). Moreover, when the subgroup analysis was performed, no statistically significant differences between groups were detected to both collagen membrane (MD: 0.78; 95%CI: -0.32 – 1.88) and acellular dermal matrix (MD: -0.50; 95%CI: -1.73 – 0.73) (Fig. 4).

-Certainty of the evidence

Table 2 shows the certainty of evidence for the GRADE approach of both meta-analyses. For both outcomes, a very low certainty of the results found was detected. This certainty of the evidence is derived from the fact that all included studies presented some concerns or high risk of bias, and a very low number of patients were included in both meta-analyses. In addition, a moderate heterogeneity was detected in the meta-analysis for horizontal bone gain at 4-5 mm of distance from the crest alveolar ridge.

**Table 2:**
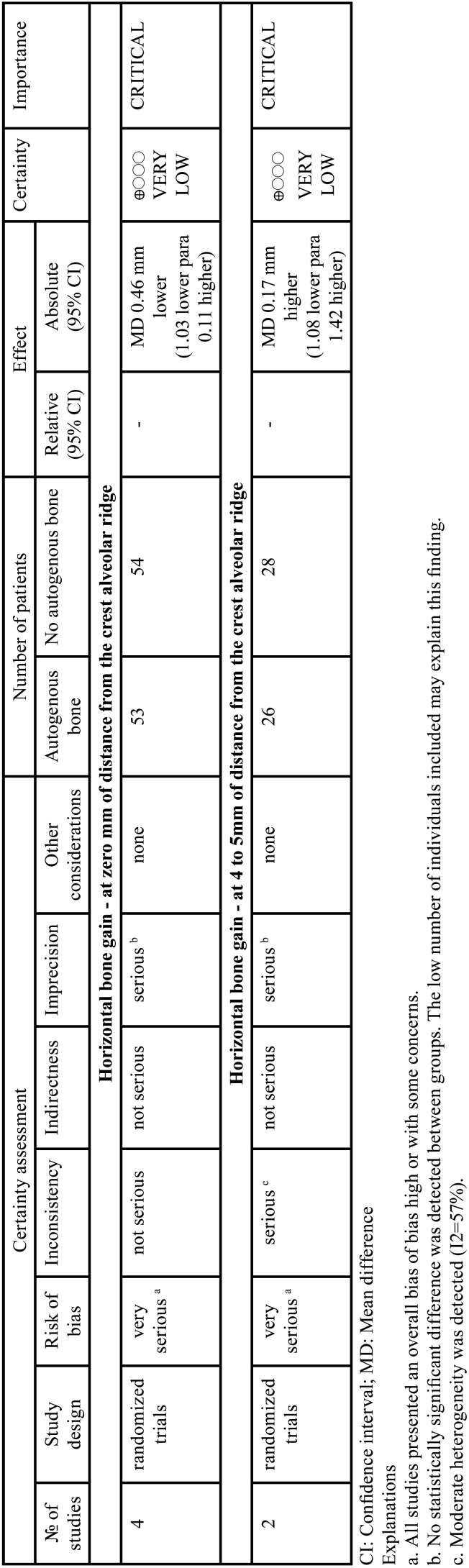
Summary of the quality assessment to all outcomes included in the meta-analyses.

## Discussion

To the best of the authors’ knowledge, this is the first systematic review and meta-analysis that has compared the clinical differences in horizontal ridge width gain between procedures performed with allogeneic bone graft alone and a combination with particulate autogenous/allograft for horizontal bone regeneration. The present study showed that the application of autogenous bone graft + allogeneic bone graft showed no significant horizontal bone gain compared with allogenous bone graft alone.

The use of particulate autologous bone graft was historically considered the gold standard for bone replacement grafts. The reason was because it contains the patient’s own cells, growth factors and biomolecules needed for osteogenesis, it has the highest degree of biological safety, biocompatibility, matched mechanical properties and scaffolding effect (29). However, the results found in this systematic review showed no statistically significant difference for horizontal bone gain, at a distance of 0 mm from the crestal alveolar ridge, and at a distance of 4 to 5 mm, between groups. As all the studies included used allogeneic bone graft, the general advantage of using this biomaterial was to provide similar mechanical properties in comparison with the autologous bone. Moreover, this graft may contain the collagenous matrix and proteins of natural bone, but it lacks viable cells (10).

Our results are in agreement with those of a randomized clinical trial that evaluated the horizontal ridge augmentation procedure with the use of guided bone regeneration with or without autogenous block grafting, after 6- (*P* = 0.26) or 18-months (*P* = 0.26) of follow-up. However, Group Autogenous block graft had a statistically significant higher prevalence of sensory disturbances (*P* = 0.02) and hematomas (*P* = 0.002) when compared with Group Guided bone regeneration (30).

Guided bone regeneration studies using both non resorbable and resorbable membranes have shown high success rates with horizontal alveolar ridge augmentation (6,31). Resorbable membranes are typically made of polyesters or tissue collagens derived from human and animal sources (31). All the studies included in this review used resorbable membranes. Two studies used collagen membrane (26,28) and the other two acellular dermal matrix (24,27), which were considered, as a subgroup, when performing the meta-analyses. Despite of that, the results showed no statistically significant difference for horizontal bone gain independently of the membrane used. Our results are in agreement with one pre-clinical study to evaluate the effectiveness of the acellular dermal matrix as a membrane for GBR, in comparison with a bioabsorbable membrane, it was concluded that acellular dermal matrix acted as a barrier in GBR, with clinical, radiographic and histomorphometric results similar to those obtained with the bioabsorbable membrane (32).

Spontaneous membrane exposure leads to decreased new bone formation (33,34). This event appears to be frequent in bone augmentation procedures. A 41.2% rate of exposure has been reported for implants placed together with non-resorbable membranes (33); however, lower incidences have also been reported (35,36). Two studies included in this systematic review showed a low rate of membrane exposure, which healed completely without loss of graft materials (26,28). This exposure occurred at only one surgical site in Group Allogeneic bone graft (28). In another study, exposure occurred in one patient in Group Allogeneic bone graft, and in four patients in Group Autogenous bone + allogeneic bone graft (*p* > 0.05) (26). Whereas in one study included in this systematic review two sites in Group Allogeneic bone, and one site in the combination group experienced early postoperative infections that resulted in near-complete loss of the grafted materials and were considered failures. This represented a rate of 12.5% infection of treatment sites, but with no statistically significant differences between groups for all studies. A systematic review by Jensen and Terheyden reported a rate of 18.9% complications with GBR procedures using resorbable membranes; however, the authors did not differentiate between infections and other complications (6).

To date, there is no consensus about the amount of autogenous bone graft needed in the guided bone regeneration procedure. In the studies included in this systematic review, different ratios of autogenous bone graft were used in combination with allogeneic bone graft, i.e., 50% / 50% (28,32) and 30% / 70% (27). One split-mouth randomized clinical study evaluated the optimal ratio of deproteinized bovine bone (DPBB) and autogenous bone (AB) for lateral augmentation procedures. They evaluated a graft mixture of 90:10 (DPBB:AB) on one side and 60:40 (DPBB:AB) on the contra lateral side. The gain in width was 5.7 mm and 6.2 mm, respectively without any significant difference between the groups (37). A similar result was reported in one prospective clinical trial, om which the researchers showed a horizontal bone gain of 5.03 mm by mixing 50% anorganic bovine bone and 50% autogenous bone. However, no control group was used in this study (38).

Two clinical studies included in this systematic review performed a histological analysis, the mean value of new bone found in the combined group was higher than that found in Group Allogeneic bone graft alone (46.07 ± 6.34% and 43.71 ± 5.63%) (28). However, the other clinical study showed that the mean value of new bone found in the combined group was lower than that found in the Group Allogeneic bone graft alone (35% and 39%) (27), nevertheless, in both studies, the differences between them were not statistically significant between the groups (*P* >0.05) (27,28). It is probably that the difference in the amount of new bone formation between the studies included in this systematic review could probably be attributed to the different time of healing between the studies (27,28) and to the ratio of autogenous bone graft used in each study 30% (27) and 50% (28). It seems that the larger the amount of autogenous bone used, the higher will be the new bone formed, which could be due to bone morphogenetic proteins in the autogenous graft and their role in osteogenesis (10). Finally, only one clinical study included in this review reported that the mean value of remaining graft particles in Group Allogeneic bone graft was higher than the value in the combined group (9.86 ± 2.16%, and 9.08 ± 2.33%, respectively), which could be due to the higher resorption rate that occurred in the autogenous bone graft, but the difference between them was not statistically significant (*P* >0.05) (28). Reports have indicated that the rates of autogenous bone graft resorption are higher, and their rate of resorption is unpredicTable, varying from 12% to 80% (10,39). Two clinical studies included in this systematic review reported the mean rate of graft resorption. In both studies the rate of resorption in Group Allogeneic bone graft + autogenous bone was higher than that of Group Allogeneic bone graft alone without statistically difference (*P* >0.05) (24,27).

Some limitations have to be taken into consideration while processing this systematic review. One of the limitations was the size of bone defects. All the studies included in this systematic review enrolled patients who had lost 2 to 4 teeth; therefore, the results of this study should be interpreted with caution and may not be generalized to larger defects. Another limitation of the present study was that the majority of the articles included showed a high risk of bias (24,26,27). Furthermore, frequently no blinding methods were applied (due to the methodological design of the studies), which increased the risk of bias. Yet another limitation could be the few studies available in the literature, which have evaluated the use of autogenous bone in combination with substitute bone graft. Within these limitations, this is the first systematic review and meta-analyses that has evaluated the use of autogenous bone in combination with allogeneic bone graft with regard to the horizontal bone gain. Some observations on the applicability of the results obtained could be formulated. The application of autogenous bone graft did not seem to lead to additional benefit in terms of horizontal bone gain when applied together with allogeneic bone graft. Further well-designed randomized clinical trials using this approach are needed to confirm these results.

The present review endeavored to summarize the best available evidence, but not always the least biased. The limitations of evidence were comprehensively summarized in a transparent manner using the GRADE approach, according to the most recent recommendations found in the Cochrane and Non-Cochrane systematic reviews 40. Further studies are warranted to increase the data of the body of evidence accumulated, considering the above-mentioned limitations.

## Conclusions

Within the limitations of this study, the collective evidence emerging from this systematic review may support the finding that the addition of autogenous bone graft to allogeneic bone graft did not significantly increase the quality and quantity of regenerated bone. The results of this review must be interpreted with caution, due to the low number of RCTs included.
